# Current infection control practices used in Australian and New Zealand cystic fibrosis centers

**DOI:** 10.1186/s12890-020-1052-y

**Published:** 2020-01-17

**Authors:** Rebecca Elizabeth Stockwell, Michelle ELizabeth Wood, Emma Ballard, Vanessa Moore, Claire Elizabeth Wainwright, Scott Cameron Bell

**Affiliations:** 1 Lung Bacteria Group, QIMR Berghofer Medical Research Institute, 300 Herston Road, Herston, QLD 4006 Australia; 2 Faculty of Medicine, The University of Queensland, Herston, QLD 4006 Australia; 3 Adult Cystic Fibrosis Center, The Prince Charles Hospital, 627 Rode Road, Chermside, QLD 4032 Australia; 4 Statistical Support Group, QIMR Berghofer Medical Research Institute, 300 Herston Road, Brisbane, QLD 4006 Australia; 5 Respiratory and Sleep Medicine, Queensland Children’s Hospital, 501 Stanley Street, South Brisbane, QLD 4101 Australia

**Keywords:** Cystic fibrosis, Infection control, Cross-infection, Contact precautions, Survey, Masks, Cleaning

## Abstract

**Background:**

The 2013 update of the Infection Prevention and Control (IP&C) Guideline outlined recommendations to prevent the spread of CF respiratory pathogens. We aimed to investigate the current infection control practices used in Australian and New Zealand (NZ) CF centers.

**Methods:**

Two online surveys were distributed to Australian and NZ CF centers regarding the uptake of selected IP&C recommendations. One survey was distributed to all the Medical Directors and Lead CF Nurses and the second survey was distributed to all the Lead CF Physiotherapists.

**Results:**

The response rate was 60% (60/100) for medical/nursing and 58% (14/24) for physiotherapy. Over 90% (55/60) of CF centers followed CF-specific infection control guidelines and consistent infection control practices were seen in most CF centers; 76% (41/54) had implemented segregation strategies for ambulatory care and no CF centers housed people with CF in shared inpatient accommodation. However, the application of contact precautions (wearing gloves and apron/gown) by healthcare professionals when reviewing a CF person was variable between CF center respondents but was most often used when seeing CF persons with MRSA infection in both ambulatory care and hospital admission (20/50, 40% and 42/45, 93% of CF centers, respectively). Mask wearing by people with CF was implemented into 61% (36/59) of centers. Hospital rooms were cleaned daily in 79% (37/47) of CF centers and the ambulatory care consult rooms were always cleaned between consults (49/49, 100%) and at the end of the clinic session (51/51, 100%); however the staff member tasked with cleaning changed with 37% (18/49) of CF centers responding that CF multidisciplinary team (MDT) members cleaned between patients whereas at the end of the clinic session, only 12% (6/51) of the CF MDT cleaned the consult room.

**Conclusions:**

Overall, Australian and NZ CF centers have adopted many recommendations from the IP&C. Although, the application of contact precautions was inconsistent and had overall a low level of adoption in CF centers. In ~ 25% of centers, mixed waiting areas occurred in the ambulatory care. Given the variability of responses, additional work is required to achieve greater consistency between centers.

## Background

The Infection Recommendations for Patients with Cystic Fibrosis (CF) were initially published in 2003 [1] and recently updated as Infection Prevention and Control (IP&C) Guideline for CF: 2013 Update [2] in response to the emerging evidence of CF respiratory pathogen transmission. The guidelines outline recommendations to reduce cross-infection in CF centers, although some of the recommendations have been debated in CF medical and lay communities [3, 4]. Furthermore, the implementation of these recommendations may be limited by logistical, resource and hospital infrastructure constraints.

Recently, the results of a US–based survey about the uptake of the IP&C guidelines focussing on education and quality improvement activities have been published [5]. The authors reported many of the updated recommendations had been adopted in US CF centers [5] however, the adoption of the IP&C recommendations outside of the US is unknown. We aimed to investigate the current infection control practices used in Australian and New Zealand CF centers including a focus on logistics of care, mask wear and cleaning.

## Methods

### Survey design

Two online surveys (www.surveymonkey.com) were developed and piloted by members of the Adult Cystic Fibrosis Center, The Prince Charles Hospital (TPCH), Australia. Survey one was distributed to all the Medical Directors and Lead Nurses of Australian and New Zealand CF centers (56 questions, Additional file 1: Figure S1). The medical/nursing survey design did not include a question about discipline information. Survey two was sent to all the Lead Physiotherapists of the same centers (41 questions, Additional file 2: Figure S2). The study was granted approval as a quality assurance activity by TPCH Human Research and Ethics Committee (HREC/17/QPCH/33). All survey questions were optional.

### Survey participants

Participants were invited via an email link to participate between September and December 2017. The surveys were distributed to 51 Center Directors (Australia, *n* = 24; New Zealand, *n* = 27), 49 Lead CF Nurses (Australia, *n* = 22; New Zealand, *n* = 27) and 24 Lead CF Physiotherapists (Australia, *n* = 23; New Zealand, *n* = 1).

### Statistical analysis

SPSS version 22 was used for statistical analysis. Data was summarised as frequency (“/ denominator”) and percentage. Comparisons of interest were between pediatric and adult treating services and between CF centres with ≤200 and > 200 patients. The center characteristics analysed from the survey responses were: center type (centers providing pediatric CF care, “pediatric-treating” and centers providing adult CF care, “adult-treating”), center size (≤200 CF patients and > 200 CF patients) and site location (Australia, New Zealand, and CF center of unknown location). Categorical variables were examined using Pearson’s Chi-squared test or Fisher’s Exact test, as appropriate, and *P* values < 0.05 were considered statistically significant.

## Results

### Medical and nursing

#### Response rates and CF center characteristics

Sixty medical/nursing responses (time to complete survey ~ 25 min) were received out of a possible 100 invited survey participants (60%). Identifying whether the response was from medical or nursing staff could not be determined due to the survey design. Furthermore, not all respondents provided site identifiers as this question was optional. As such, all responses to the medical/nursing survey (*n* = 60) were analysed and reported below.

##### CF centre type

Thirty-eight of 60 (63%) were paediatric-treating (23 provided paediatric services only and 15 provided combined paediatric and adult services) and 22 (37%) were adult-treating CF centres.

##### CF centre location

Twenty (33%) responses were Australian, 14 (23%) were New Zealand and 26 (43%) were CF centres of unknown location.

##### CF centre size

Fifty-two (87%) responses were from CF centres with ≤200 patients while the remaining 8 (13%) were from CF centres with > 200 patients. Centre sizes differed between Australia and New Zealand with predominately (86%) small centres (*p* < 0.001). Differences between Australia, New Zealand and those of unknown location servicing ≤200 patients were examined and as no differences were identified with the exception of contact precautions during hospital admission and ambulatory care, further analysis between CF centres with ≤200 and > 200 patients was considered appropriate.

#### Infection control policies in CF centers

Overall, 92% (55/60) of medical/nursing respondents followed CF-specific infection control guidelines. Response rates were similar between center type (*p* = 0.23) and center size (*p* = 1.00) (Table 1).

**Table 1:** Infection control strategies used

	Pediatric-treating services (*n* = 38)	Adult-treating services (*n* = 22)	*p*-value	≤200 patients (*n* = 52)	> 200 patients (*n* = 8)	*p*-value
Implementation of CF-specific infection control policy, n (%)	37/38 (97%)	18/22 (82%)	0.23	47/50 (94%)	8/8 (100%)	1.00
Contact precautions						
During hospital admission:				NA	NA	NA
All people with CF, n (%)	6/19 (32%)	8/9 (89%)	0.013			
MRSA infection, n (%)	26/29 (90%)	16/16 (100%)	0.54			
MRO infection, n (%)	15/21 (71%)	11/12 (92%)	0.22			
During ambulatory care:				NA	NA	NA
All people with CF, n (%)	4/32 (13%)	4/18 (22%)	0.44			
MRSA infection, n (%)	13/32 (41%)	7/18 (39%)	0.90			
MRO infection, n (%)	9/26 (35%)	6/18 (33%)	0.93			
Patient mask wearing policy	24/37 (65%)	12/22 (55%)	0.43	29/51 (57%)	7/8 (88%)	0.13
Use of face-masks (regardless of whether mask-policy implemented):			1.00			0.25
Surgical mask, n (%)	22/30 (73%)	11/14 (79%)		27/37 (73%)	6/7 (86%)	
N95 mask, n (%)	2/30 (7%)	1/14 (7%)		2/37 (5%)	1/7 (14%)	
Combination of mask types/other mask types, n (%)	6/30 (20%)	2/14 (14%)		8/37 (22%)	0/7 (0%)	
Segregation strategies						
Hospital rooms used:			0.69			0.21
Single room with ensuite only^^^, n (%)	19/31 (61%)	10/18 (56%)		27/43 (63%)	2/6 (33%)	
Combination of room types, n (%)	12/31 (39%)	8/18 (44%)		16/43 (37%)	4/6 (67%)	
During ambulatory care:			0.51			0.38
Segregated waiting areas, n (%)	28/35 (80%)	13/19 (68%)		36/46 (78%)	5/8 (63%)	
Communal waiting areas, n (%)	7/35 (20%)	6/19 (32%)		10/46 (22%)	3/8 (38%)	

*CF* cystic fibrosis, *MRO* multi-drug resistant, *MRSA* methicillin-resistant *Staphylococcus aureus*; ^^^includes isolation rooms also (*n* = 1 response), *NA* not applicable

#### Contact precautions

##### Inpatient care

CF center respondents varied in their approach of healthcare professionals applying contact precautions (gloves and gown/apron) during review of inpatients with CF, according to respiratory microbiological infection status. Of the CF center respondents, 50% (14/28) applied contact precautions when reviewing inpatients regardless of infection type, 93% (42/45) applied contact precautions when seeing a CF inpatient with MRSA infection and 79% (26/33) applied contact precautions when seeing a CF inpatient with multi-drug resistant organism (MRO) infection (*Burkholderia cepacia* complex and *Mycobacterium abscessus* group). Almost 90% of adult-treating CF healthcare professionals applied contact precautions when reviewing all people with CF compared to 32% of pediatric centers (*p* = 0.013) (Table 1). The uptake of healthcare professionals applying contact precautions was similar between respondents from pediatric-treating and adult-treating CF centers when reviewing a CF inpatient with MRSA or MRO infections (Table 1). In CF centers with ≤200 patients, healthcare professionals applied contact precautions in 89% of Australian centers, 40% of New Zealand centers and 20% of centers with unknown location when seeing all inpatients with CF (Table 2). Similarly, responses from CF centers with ≤200 patients, 100% of Australian centers, 86% of New Zealand centers and 60% of centers with unknown location reported healthcare professionals applying contact precautions when seeing CF inpatients with MRO infection. The uptake of healthcare professionals following contact precautions when reviewing inpatients with MRSA infection was similar between all locations (Australia, New Zealand or unknown location) in CF centers with ≤200 patients.

**Table 2:** Use of contact precautions by CF center location (in CF centers ≤200 patients)

	Australia (*n* = 15)	New Zealand (*n* = 14)	Not specified(*n* = 23)	*p*-value
Contact precautions				
During hospital admission:				
All people with CF, n (%)	8/9 (89%)	2/5 (40%)	2/10 (20%)	0.007
MRSA infection, n (%)	14/14 (100%)	9/10 (90%)	14/15 (93%)	0.52
MRO infection, n (%)	11/11 (100%)	6/7 (86%)	6/10 (60%)	0.030
During ambulatory care:				
All people with CF, n (%)	5/13 (38%)	0/13 (0%)	2/16 (13%)	0.031
MRSA infection, n (%)	10/13 (77%)	3/13 (23%)	6/16 (38%)	0.016
MRO infection, n (%)	10/12 (83%)	1/10 (10%)	2/14 (14%)	< 0.001

*CF* cystic fibrosis, *MRSA* methicillin-resistant *Staphylococcus aureus, MRO* multi-resistant organism (*Burkholderia cepacia* complex; *Mycobacterium abscessus* group)

##### Ambulatory care

Contact precautions were applied by healthcare professionals in 16% (8/50) of CF centers respondents when seeing all people with CF, 40% (20/50) of CF center respondents when seeing a CF person with MRSA infection and 34% (15/44) of CF center respondents when seeing a CF person with MRO infection. The use of contact precautions by healthcare professionals was similar between pediatric- and adult-treating CF centers for all people and those with MRSA or MRO infections (Table 1). In CF centers with ≤200 patients, the use of contact precautions was significantly different between site location (country) (Table 2). During ambulatory care, 38% of Australian CF centers, 0% of New Zealand centers and 13% of centers with unknown location applied contact precautions when reviewing all people with CF. During ambulatory review of people with CF and MRSA infection, 77% of Australian CF center, 23% of New Zealand centers and 38% of centers with unknown location applied contact precautions. During ambulatory review of people with CF and MRO infection, 83% of Australian CF centers, 10% of New Zealand centers and 14% of centers with unknown location applied contact precautions.

#### Segregation of people with cystic fibrosis during hospital visits

##### Inpatient accommodation

Segregation of people with CF during hospital admissions was universal, with all CF centers responding that inpatients with CF did not share the same room. The types of hospital rooms used during admission were similar between center types and center sizes (Table 1) with 59% (29/49) of respondents admitting people with CF to a single room with ensuite.

##### Ambulatory care waiting areas

Seventy-six percent (41/54) of CF center respondents used segregation strategies at ambulatory care clinics (Table 1), with 61% (33/54) of respondents directing CF patients to their designated consult room on arrival. Communal waiting areas were used by 24% (13/54) of CF center respondents. The waiting area types were similar between CF center type and center size (Table 1).

#### Patient mask-wear policy

Sixty-one percent (36/59) of CF center respondents had implemented a mask wear policy for people with CF when in communal areas of the hospital. Implementation of patient mask-wear policies were similar between center type and center size (Table 1). The mask type was similar across the center type and center size (Table 1). Surgical masks were used in 75% (33/44) of CF centers, N95 masks used in 7% (3/44) CF centers and other masks types or a combination of masks were used in 18% (8/44) CF centers (Table 1).

#### Cleaning

##### Inpatient rooms

Inpatient rooms were cleaned daily in 79% (37/47) of CF center respondents. The frequency of patient hospital room cleaning was similar between CF center type and center size (Table 3).

**Table 3:** Cleaning of CF ambulatory clinics and inpatient rooms

	All CF centers (*n* = 60)	Pediatric-treating services (*n* = 38)	Adult-treating services (*n* = 22)	*p*-value	≤200 patients (*n* = 52)	> 200 patients (*n* = 8)	*p*-value
Frequency of room cleaning:				0.24			1.00
Daily, n (%)	37/47 (79%)	25/29 (86%)	12/18 (67%)		32/42 (76%)	5/5 (100%)	
Not daily, n (%)	6/47 (13%)	2/29 (7%)	4/18 (22%)		6/42 (14%)	0/5 (0%)	
Don’t know, n (%)	4/47 (9%)	2/29 (7%)	2/18 (11%)		4/42 (10%)	0/5 (0%)	
Cleaning of ambulatory rooms							
Between patients:				0.61			0.23
CF MDT, n (%)	18/49 (37%)	10/31 (32%)	8/18 (44%)		15/41 (37%)	3/8 (38%)	
Clinic nursing staff, n (%)	20/49 (41%)	12/31 (39%)	8/18 (44%)		17/41 (41%)	3/8 (38%)	
Cleaning staff, n (%)	4/49 (8%)	3/31 (10%)	1/18 (6%)		2/41 (5%)	2/8 (25%)	
Other staff^~^, n (%)	7/49 (14%)	6/31 (19%)	1/18 (6%)		7/41 (17%)	0/8 (0%)	
At end of clinic session:				0.47			0.41
CF MDT, n (%)	6/51 (12%)	3/33 (9%)	3/18 (17%)		4/43 (9%)	2/8 (25%)	
Clinic nursing staff, n (%)	10/51 (20%)	7/33 (21%)	3/18 (17%)		9/43 (21%)	1/8 (13%)	
Cleaning staff, n (%)	31/51 (61%)	19/33 (58%)	12/18 (67%)		27/43 (63%)	4/8 (50%)	
Other staff^~^, n (%)	4/51 (8%)	4/33 (12%)	0/18 (0%)		3/43 (7%)	1/8 (13%)	

*CF* cystic fibrosis, *MDT* multidisciplinary team

##### Ambulatory clinics

All CF center respondents (49/49) reported their ambulatory clinic rooms were cleaned between each CF patient consultations by a hospital staff member and this was similar between center type and center size (Table 3). Specifically, the cleaning was performed by members of the CF multidisciplinary team (MDT) or clinic nursing staff in 78% (38/49) of CF centers and by cleaning staff in 8% (4/49) of CF centers. At the end of the CF clinic session, all CF center respondents (51/51) reported their clinic rooms were always cleaned by a hospital staff member. The staff member tasked with cleaning was similar between the center types and center size (Table 3). At the end of the ambulatory care session, cleaning staff cleaned the consultation rooms in 61% (31/51) of CF centers and CF MDT or clinic nursing staff cleaned the consultation rooms in 31% (16/51) of CF centers.

### Physiotherapy respondents

#### Response rates and CF center characteristics

Fourteen physiotherapy responses (time to complete survey ~ 20 min) were received from the 24 invited survey participants (58%): nine of 14 (64%) were pediatric-treating (5 provided pediatric services only and 4 provided combined pediatric and adult services) and 5 (36%) were adult-treating CF centers.

#### Contact precautions

##### Infection control in the gym

The types of gym sessions used (individual or group sessions) were similar between pediatric- and adult-treating CF center respondents (Table 4). Individual sessions were used in 93% (13/14) CF centers (Table 4). The gym was always cleaned between patients and the staff member tasked with cleaning was similar between pediatric- and adult-treating CF center respondents (Table 4). In 43% (6/14) of CF centers, the gym was cleaned by a physiotherapist only, whereas in 57% (8/14) of CF centers, the gym was cleaned by physiotherapists and other staff (e.g. Allied Health Assistants, cleaning staff). In one CF center (7%), the patients with CF were encouraged to clean the gym equipment.

**Table 4:** Infection control strategies used: physiotherapy responses

	Pediatric-treating services (*n* = 9)	Adult-treating services (*n* = 5)	*p*-value
Segregation strategies			
Gym scheduling:			1.00
Individual sessions, n (%)	8/9 (89%)	5/5 (100%	
With other patients (without CF), n (%)	1/9 (11%)	0/5 (0%)	
Gym cleaning procedures			
Between patients:			0.58
Physiotherapy staff only, n (%)	3/9 (33%)	3/5 (60%)	
Physiotherapy and other^&^ staff, n (%)	6/9 (67%)	2/5 (40%)	

*CF* cystic fibrosis; ^&^Allied Health Assistants, cleaning staff, patients

## Discussion

The key finding from our study was that almost all respondents from CF centers in Australia and New Zealand had implemented and followed CF-specific infection control guidelines based predominantly on the current IP&C recommendations [2]. In a recent survey of US CF centers, half of the centers had adopted ≥75% of the selected IP&C recommendations [5]. While the two surveys (ours and the US) had differing approaches, the key message is that many of the Australian, New Zealand and US CF-centers have implemented infection control strategies based upon the published IP&C recommendations [2]. The adoption of IP&C recommendations surveyed in Australian and New Zealand CF centers were similar between center type (pediatric-treating vs adult-treating) and center size (≤200 patients vs > 200 patients), which was similar to the results of the US survey [5]. Regional differences in the adoption of IP&C recommendations were found in the US-based survey [5]; however, we did not analyse the regional differences due to the very different healthcare models used between the two countries: Australia has 24 specialised CF centers that care for > 3400 people with CF [6] whereas New Zealand has 27 specialised CF centers that care for ~ 500 people with CF [7].

Physical barriers were outlined in the current IP&C to prevent cross-infection in CF centers such as the use of contact precautions and segregation of people with CF during hospital visits [2]. Contact precaution use was similar between CF center type when reviewing admitted and ambulatory care patients, yet an important finding was the overall low rates for the ambulatory care setting, and that this was greatest in the New Zealand based centers. One exception was that adult-treating CF center respondents applied contact precautions more often when reviewing all CF inpatients compared to their pediatric counterparts. The use of contact precautions in CF centers with ≤200 patients was also more variable. Australian CF center respondents were more likely to follow contact precautions both when reviewing hospitalized and ambulatory care patients. However, reviewing CF inpatients with MRSA infection was an exception and contact precaution use was similar across the different locations; most likely due to established guidelines when caring for any inpatient with MRSA infection [8]. The smaller CF centers and New Zealand centers may not follow the application of contact precautions during ambulatory clinics due to a perceived view of low cross-infection risk; for example, one person with CF may attend an ambulatory clinic session. Overall, the use of contact precautions in Australian and New Zealand CF centers was less common compared to the rates in US CF centers which reported 90% of centers use contact precautions when reviewing all people with CF (inpatient and ambulatory care settings) [9].

The segregation of people with CF during hospital visits (both inpatient and ambulatory care settings) was adopted in the majority of Australian and New Zealand CF centers despite it being costly and logistically challenging. The use of single rooms with ensuites for inpatient accommodation was lower in Australian and New Zealand CF centers (~ 60%) compared with German CF centers where 81% of CF inpatients were accommodated in a single room with an adjacent bathroom [10]. Most importantly, no CF center respondents in our study reported people with CF sharing inpatient rooms with another person with CF. Though in the ambulatory care setting, one-quarter of the CF center respondents continued to use shared waiting areas without formal segregation. Prior studies have demonstrated that enhanced cohort segregation has led to reduced rates of shared strain *Pseudomonas aeruginosa* infection [11–14]. Our results highlight the potential opportunities for cross-infection in a proportion of CF centers using shared ambulatory care waiting areas.

To prevent cross-infection in CF centers, face masks are recommended to be worn by people with CF to reduce dispersion of CF respiratory pathogens [2]. The majority (61%) of Australian and New Zealand CF center respondents had implemented a patient mask-wear policy. While this rate is similar to a recently published study of German CF centers where 60% supplied masks at the entrance to the ambulatory clinics [10], a US study reported patient-mask wear adoption rates of 85% in inpatient and 87% in ambulatory settings [9]. The low adoption rates of patient-mask wear policies in Australian and New Zealand CF centers is surprising considering hospitals almost universally require people with respiratory illness symptoms to wear a surgical mask while in a hospital facility [8]. However, patient-mask wear policies have been a contentious issue in the CF community [15], which may point to variable uptake of mask wear in CF centers. For those respondents which had implemented a patient-mask wear policy, surgical masks were the most common form of source control used in Australian and New Zealand CF centers. While the IP&C recommendations do not recommend that respirators be worn by people with CF as source control [2], a small number (< 10%) of Australian and New Zealand CF centers did use N95 masks as source control. The low uptake of N95 masks as source control is likely due to the poor comfort of N95 masks reported by people with CF [16, 17].

The cleanliness of the hospital environment, frequency of cleaning such as daily cleaning of inpatient rooms [18–20] and cleaning of ambulatory care settings [21] has been reported to reduce hospital cross-infections. The implementation of cleaning procedures into CF centers is recommended to prevent CF pathogen acquisition [2]. The majority of CF center respondents from Australia and New Zealand cleaned inpatient rooms on a daily basis and most CF centers cleaned ambulatory care consult rooms both between patients and at the end of the clinic session. In the US survey, more than three-quarters of the ambulatory care rooms were reported to be cleaned [5] although the timing of the cleaning protocol (e.g. between patients, at the end of the day, etc) was not reported.

Our study also surveyed CF physiotherapists about the uptake of the IP&C recommendations especially the delivery of exercise sessions, an integral component of CF treatment [22]. Physiotherapy respondents delivered gym-based exercise mostly as individual patient sessions. Furthermore, the gyms were cleaned between every patient and the physiotherapist was most often involved in the cleaning (either solely or in combination with other staff). Interestingly, one CF center respondent that the patient assisted with cleaning of the gym equipment and this supports the findings of an earlier study that reported engagement of people with CF may enhance patient adherence to infection control measures [23].

The impact on people with CF and their families when implementing these enhanced IP&C recommendations in CF centers [2] must also be considered. The most recent IP&C guidelines included consultation with three parents of children with CF and an adult with CF along with CF healthcare experts [2]. While patient engagement in developing and implementing changes to IP&C guidelines should remain a priority, the knowledge levels in the wider CF community can vary and ongoing education of the CF community should also be prioritised. Furthermore, the debate in the medical community around appropriate infection control measure to implement in CF centers [3, 4] and the lack of understanding of how CF respiratory pathogens are acquired can heighten anxiety in people with CF and their families [24, 25]. Some recommendations are accepted into the CF community despite the associated negative consequences. For example, segregation is supported in the CF community, although the psychosocial effects of segregating patients can be considerable [26, 27].

There were a number of limitations to this study. Firstly, all survey questions were optional (including the identifying questions) to enhance honest survey responses which may have resulted in frank feedback about local infection control policies and practices. The same survey was sent to Center Directors’ and Lead Nurses’ with no identifier to differentiate which discipline responded (medical or nursing). Additionally, the site identifier was an optional question and the majority of medical/nursing respondents did not answer or did not provide sufficient details to identify the individual site. The bias due to responses from both parties on key outcomes is unknown as we cannot assume both parties responded for each CF center. Interpretation of results for center type or center size comparisons has thus always been in reference to the responses reported for each category. Secondly, only 60% of invited survey participants responded to the survey which may influence the generalisability of the survey results to all CF centers [28]. Thirdly, Australian and New Zealand CF centers function with very different models of care and while care has been taken to remove these influencing factors, it is unable to be eliminated from the data collection, analysis, and interpretation and may have biased the survey results. Finally, the impact of implementing the IP&C recommendations into CF centres on people with CF and their families has not been evaluated here and requires additional study, yet is an important aspect in effectively implementing the IP&C recommendations [5].

## Conclusion

In conclusion, Australian and New Zealand CF centers who responded to this survey have readily adopted many of the IP&C recommendations and established some consistent infection control strategies. However, the surveys identified areas of inconsistencies between CF centers (e.g., following contact precautions), and highlights opportunities for improvements. Ensuring the implementation of consistent IP&C recommendations into CF centers is desirable, as this may reduce the risk of cross-infection between people with CF and will likely facilitate the transitioning of care to other CF centers.

## Supplementary information

**Additional file 1: Figure S1.** Medical/nursing infection control survey.

**Additional file 2: Figure S2**. Physiotherapy infection control survey.

## Data Availability

The datasets used and/or analysed during the current study are available from the corresponding authors on reasonable request.
