# Supplementary material for: Current infection control practices used in Australian and New Zealand cystic fibrosis centers
Source: BMC Pulm Med. 2020 Jan 17;20:16. doi: 10.1186/s12890-020-1052-y (PMC6969421; doi:10.1186/s12890-020-1052-y)
Supplement: Supplementary file 1 — Additional file 1: Figure S1. Medical/nursing infection control survey. [file 12890_2020_1052_MOESM1_ESM.pdf]

Medical and nursing infection control

Demographics

1. What is your Australian CF data registry site number?

2. What CF service does your hospital provide?

- Adult
- Paediatric
- Combined adult and paediatric

3. How many people with CF attend your service?

- <50
- 50-100
- 100-200
- 200-300
- >300

4. On average, how many people with CF are admitted to your hospital on a weekday?

- <1
- 1-3
- 4-6
- 7-10
- 11-15
- 16-20
- >20

Medical and nursing infection control

Infection control policy implementation

Please answer the following questions about infection control policies that existed in your centre as at 31st July 2017.

5. Has your centre changed infection control practices in the last 12 months?

- ◯ Yes
- ◯ No

If yes, what date did your centre implement the changes?

6. Have you changed infection control practices for outpatient care?

- ◯ Yes
- ◯ No
- ◯ Not applicable

If yes, please specify the changes your centre has made

7. Do you plan to change your infection control practices in the next 12 months?

- ◯ Yes
- ◯ No

8. Have you changed infection control practices for inpatient care?

- ◯ Yes
- ◯ No
- ◯ Not applicable

If yes, please specify the changes your centre has made

9. Has there been a change to the cleaning procedures?

- ◯ Yes
- ◯ No
- ◯ Not applicable

If yes, please specify the changes your centre has made

10. Have you implemented a mask wear policy?

- ◯ Yes
- ◯ No
- ◯ Not applicable

If yes, please specify the changes your centre has made

Medical and nursing infection control

General CF infection control guidelines

Please answer the following questions about infection control policies that existed in your centre as at 31st July 2017.

11. Are you currently following infection control guidelines for managing people with CF?

- ○ Yes
- ○ No

12. If yes, which guidelines do you follow (tick all that apply)?

- ☐ Australian CF Infection Control guidelines
- ☐ US CFF Infection Control guidelines
- ☐ Local hospital infection control guidelines
- ☐ Other, please state below

If other, please specify

13. Are there any specific infection control guidelines for managing people with CF infected with the following (please tick all that apply)?

- ☐ No Pseudomonas
- ☐ *Pseudomonas aeruginosa*
- ☐ MRSA
- ☐ *Burkholderia cepacia* complex
- ☐ *Mycobacterium abscessus* complex
- ☐ NTM species other than *M. abscessus* complex
- ☐ Others, please state below
- ☐ If other, please specify

14. How often are people with CF screened for bacterial respiratory infections (please tick all that apply)?

- [ ] When sputum available
- [ ] Each clinic visit
- [ ] Each inpatient admission
- [ ] Every 3 months
- [ ] Every 6 months
- [ ] Every year
- [ ] Other, please state below
- [ ] If other, please specify

15. When is a person with CF no longer considered infected with specific organisms?

	Never	Number of negative cultures	Time since last cultured
Pseudomonas aeruginosa	○	○	○
MRSA	○	○	○
Burkholderia cepacia complex	○	○	○
Mycobacterium abscessus complex	○	○	○
NTM species other than *M. abscessus* complex	○	○	○

Please specify the number of negative cultures or the time since culture below:

16. If no longer considered infectious, can people with CF return to an outpatient clinic appropriate to the patients' current microbiology?

- ○ Yes
- ○ No

If yes, please specify how this occurs

Medical and nursing infection control

Infection control in the communal areas of the hospital

17. Where is lung function testing performed for people with CF infected with:

	Clinic rooms	Communal room in respiratory laboratory	Separate room in respiratory laboratory	Other
No Pseudomonas	◯	◯	◯	◯
Pseudomonas aeruginosa	◯	◯	◯	◯
MRSA	◯	◯	◯	◯
Burkholderia cepacia complex	◯	◯	◯	◯
Mycobacterium abscessus complex	◯	◯	◯	◯
NTM species other than *M. abscessus* complex	◯	◯	◯	◯

If other, please specify location

18. If lung function is performed in the respiratory lab (lung function lab), where do people with CF sit while waiting?

◯ Communal respiratory lab waiting area

◯ Clinic room

◯ Other, please state below

If other, please specify

19. Is there advice given to people with CF (and their carers) about what to do in communal areas of the hospital during outpatient visits?

◯ Yes

◯ No

Comments

Medical and nursing infection control

Infection control at CF outpatient clinics

20. Where do people with CF sit while waiting for outpatient consultation?

- ○ Communal waiting area
- ○ In designated consulting rooms on arrival
- ○ Away from clinic with a call system (e.g. text message, page system)
- ○ Other

If other, please specify

21. Are people with CF assigned a clinic room for all consultations to take place in during outpatient visit (i.e. medical, nursing and allied health go to patient room)?

- ○ Yes
- ○ No

22. Is there a cleaning procedure implemented for cleaning outpatient clinic rooms (please tick all that apply)?

	Yes	No
All people with CF	○	○
People with CF and specific respiratory infections	○	○

If yes, what is the cleaning procedure?

23. Is there a dedicated infectious outpatient clinic for people with CF?

- ○ One infection clinic with different infections (e.g. MRSA, *Mycobacterium abscessus* complex, *Burkholderia cepacia* complex, etc)
- ○ Multiple 'sole' infection special clinics

24. What personal protective equipment do staff wear during infectious outpatient clinics (please tick all that apply)?

	Gloves	Apron	Gown	Surgical mask with ties	Surgical mask with elastic ear loops	N95 respirator	Other	None
No Pseudomonas	☐	☐	☐	☐	☐	☐	☐	☐
Pseudomonas aeruginosa	☐	☐	☐	☐	☐	☐	☐	☐
MRSA	☐	☐	☐	☐	☐	☐	☐	☐
Burkholderia cepacia complex	☐	☐	☐	☐	☐	☐	☐	☐
Mycobacterium abscessus complex	☐	☐	☐	☐	☐	☐	☐	☐
NTM species other than *M. abscessus* complex	☐	☐	☐	☐	☐	☐	☐	☐

If other, please specify

25. Is there a washout period in specific rooms between patient consultations during outpatient clinics (please tick all that apply)?

	No washout	<30 minutes	30-60 minutes	>60 minutes
No Pseudomonas	○	○	○	○
Pseudomonas aeruginosa	○	○	○	○
MRSA	○	○	○	○
Burkholderia cepacia complex	○	○	○	○
Mycobacterium abscessus complex	○	○	○	○
NTM species other than *M. abscessus* complex	○	○	○	○

26. Are extra cleaning procedures implemented after the infectious outpatient clinic is completed (please tick all that apply)?

- [ ] MRSA
- [ ] *Burkholderia cepacia* complex
- [ ] *Mycobacterium abscessus* complex
- [ ] NTM species other than *M. abcessus* complex
- [ ] Other

If other, please specify

27. Who cleans the outpatient rooms *between* patient consultations?

- ○ CF team member
- ○ Nursing staff
- ○ Cleaning staff
- ○ Not cleaned
- ○ Other (please specify)

28. Do you change the bed linen in the outpatient rooms *between* patient consultations?

- ○ Yes
- ○ No
- ○ Bed linen not used

29. What is cleaned in outpatient rooms *between* patient consultations (please tick all that apply)?

	Alcohol-based wipes	Biocidal wipes	Sporocidal wipes	Other cleaning product	Not cleaned	Don't know
Desk	☐	☐	☐	☐	☐	☐
Chairs	☐	☐	☐	☐	☐	☐
Bed	☐	☐	☐	☐	☐	☐
Door handle	☐	☐	☐	☐	☐	☐
Medical equipment	☐	☐	☐	☐	☐	☐
Computer keyboard	☐	☐	☐	☐	☐	☐
Other	☐	☐	☐	☐	☐	☐

For other cleaning product or other in room item, please specify

30. Who cleans the outpatient rooms at the *end* of the clinic session?

- ○ CF team member
- ○ Nursing staff
- ○ Cleaning staff
- ○ Not cleaned
- ○ Other (please specify)

31. Do you change the bed linen in the outpatient rooms at the *end* of the clinic session?

- ○ Yes
- ○ No
- ○ Bed linen not used

32. What is cleaned in outpatient rooms at the *end* of the clinic session (please tick all that apply)?

	Alcohol-based wipes	Biocidal wipes	Sporocidal wipes	Other cleaning product	Not cleaned	Don't know
Desk	☐	☐	☐	☐	☐	☐
Chairs	☐	☐	☐	☐	☐	☐
Bed	☐	☐	☐	☐	☐	☐
Door handle	☐	☐	☐	☐	☐	☐
Medical equipment	☐	☐	☐	☐	☐	☐
Computer keyboard	☐	☐	☐	☐	☐	☐
Other	☐	☐	☐	☐	☐	☐

For other cleaning product or other in room item, please specify

Medical and nursing infection control

Infection control during hospital admissions

33. How are people with CF managed during a hospital admission?

- [ ] Dedicated CF ward
- [ ] General respiratory ward(s)
- [ ] Infectious ward(s)
- [ ] General medical ward(s)
- [ ] Other

If other, please specify

34. What accommodation is available for people with CF during hospitalisation (please tick all that apply)?

- [ ] Single room with ensuite
- [ ] Single room with shared bathroom
- [ ] Multi-bed room with other people with CF
- [ ] Multi-bed room without other people with CF
- [ ] Isolation room (e.g. negative pressure room)
- [ ] Other

If other, please specify

35. Do you have a washout period in the patient rooms between patient stays (please tick all that apply)?

- [ ] No washout
- [ ] <30 minutes
- [ ] 30-60 minutes
- [ ] >60 minutes

36. Where is physiotherapy (excluding exercise) performed on people with CF during hospital admission (please tick all that apply)?

- [ ] Inpatient room
- [ ] CF gym
- [ ] General gym
- [ ] Other

If other, please specify

37. Where is exercise performed during hospital admission (please tick all that apply)?

- [ ] Inpatient room
- [ ] CF gym
- [ ] General gym
- [ ] Outdoors/open areas
- [ ] Other

If other, please specify

38. Are patients scheduled for gym attendance?

- ○ Yes
- ○ No

If yes, please specify

39. Is there a separate gym for infectious patients?

- ○ Yes
- ○ No

40. Is individual observation equipment available for each inpatient?

	Yes	No
Blood pressure monitor	○	○
Spirometer	○	○
Thermometer	○	○
Oximeter	○	○
Other	○	○

If other, please specify

41. Where is spirometry performed during hospital admission (please tick all that apply)?

	Patient's own hospital room	Communal room in respiratory lab	Separate room in respiratory lab	Other (e.g. uses personal spirometer)
No Pseudomonas	◯	◯	◯	◯
Pseudomonas aeruginosa	◯	◯	◯	◯
MRSA	◯	◯	◯	◯
Burkholderia cepacia complex	◯	◯	◯	◯
Mycobacterium abscessus complex	◯	◯	◯	◯
NTM species other than *M. abscessus* complex	◯	◯	◯	◯

Comments

42. Are staff provided any personal protective equipment when entering patient rooms (please tick all that apply)?

	Gloves	Apron	Gown	Surgical mask with ties	Surgical mask with elastic ear loops	N95 respirator	Other
No Pseudomonas	☐	☐	☐	☐	☐	☐	☐
Pseudomonas aeruginosa	☐	☐	☐	☐	☐	☐	☐
MRSA	☐	☐	☐	☐	☐	☐	☐
Burkholderia cepacia complex	☐	☐	☐	☐	☐	☐	☐
Mycobacterium abscessus complex	☐	☐	☐	☐	☐	☐	☐
NTM species other than *M. abscessus* complex	☐	☐	☐	☐	☐	☐	☐

If other, please specify

43. Do other members of the multidisciplinary team (e.g. social worker, dietician, etc) follow the same infection control procedures when reviewing inpatients?

◯ Yes

◯ No

◯ Don't know

◯ If no, what is different?

44. How frequently are patient rooms cleaned during admission?

- ◯ Daily
- ◯ Every second day
- ◯ Weekly
- ◯ At end of admission
- ◯ Other (please specify)

45. Who cleans the areas in the patient rooms (please tick all that apply)?

	Cleaning staff	Nursing staff	Other	Not cleaned
Bed	☐	☐	☐	☐
Handrails	☐	☐	☐	☐
Cupboards/benches	☐	☐	☐	☐
Door handles	☐	☐	☐	☐
Medical equipment	☐	☐	☐	☐
Patient bathroom	☐	☐	☐	☐
Other	☐	☐	☐	☐

For other in room item or other personnel, please specify

46. How is the patient room cleaned (please tick all that apply)?

	Alcohol based wipes	Biocidal wipes	Sporocidal wipes	Other cleaning product	Not cleaned
Bed	☐	☐	☐	☐	☐
Handrails	☐	☐	☐	☐	☐
Cupboards/benches	☐	☐	☐	☐	☐
Door handles	☐	☐	☐	☐	☐
Medical equipment	☐	☐	☐	☐	☐
Bathrooms	☐	☐	☐	☐	☐
Other	☐	☐	☐	☐	☐

For other cleaning product or other in room item, please specify

47. Do you change the curtains between patient admissions?

- ◯ Yes
- ◯ No
- ◯ We don't use curtains

Medical and nursing infection control

Mask wearing for source control

48. Are face masks advised for people with CF?

- ◯ Yes
- ◯ No

49. What type of face masks are people with CF required to wear (tick all that apply)?

- ☐ Surgical mask with ties
- ☐ Surgical mask with elastic ear loops
- ☐ N95 respirator
- ☐ Other

If other, please specify

50. Do you give instructions on how to wear the mask?

	Yes	No
Where masks are located?	◯	◯
How to apply a mask?	◯	◯
How long to wear a mask?	◯	◯
When to change a mask?	◯	◯
How to dispose of masks?	◯	◯
Hand hygeine after touching the mask?	◯	◯
Hand hygeine after removing the mask?	◯	◯

51. Who applies the mask?

- ◯ Self
- ◯ Carer
- ◯ Healthcare worker

52. Are people with CF and/or carers trained in applying a mask?

	Yes	No
Signage	◯	◯
One-on-one training	◯	◯
Video	◯	◯

Comments

53. During outpatient visits, where must the mask be worn (please tick all that apply)?

- ☐ All parts of the hospital
- ☐ All parts of the hospital excluding the clinic room allocated
- ☐ Visiting lung function
- ☐ Visiting pharmacy
- ☐ Walking in corridors
- ☐ Other
- ☐ If other, please specify

54. Are people with CF requested to wear a mask during the following procedures?

	Optional	Required
Exercise in the gym	◯	◯
Physiotherapy	◯	◯
Other	◯	◯

If other, please specify

55. During hospital admission, are people with CF required to wear masks outside their hospital room?

- ◯ Yes
- ◯ No

If yes, please specify

56. Are there any other infection control procedures not highlighted in the above questions, that you are required to undertake when treating people with certain infections?
