# Supplementary material for: Current infection control practices used in Australian and New Zealand cystic fibrosis centers
Source: BMC Pulm Med. 2020 Jan 17;20:16. doi: 10.1186/s12890-020-1052-y (PMC6969421; doi:10.1186/s12890-020-1052-y)
Supplement: Supplementary file 2 — Additional file 2: Figure S2. Physiotherapy infection control survey. [file 12890_2020_1052_MOESM2_ESM.pdf]

Physiotherapy infection control

Demographics

1. What is your Australian CF data registry site number?

2. What CF services does your hospital provide?

- Adult
- Paediatric
- Combined adult and paediatric services

3. On average, how many people with CF does the physiotherapy team consult each day (total of inpatient and outpatients):

- <5
- 5-10
- 10-20
- >20

Physiotherapy infection control

Infection control policy implementation

Please answer the following questions about infection control policies that existed in your centre as at 31st July 2017.

4. Has your centre changed infection control practices in the last 12 months?

- ◯ Yes
- ◯ No

If yes, what date did your centre implement the changes?

5. Have you changed infection control practices for outpatient care?

- ◯ Yes
- ◯ No
- ◯ Not applicable

If yes, please specify the changes your centre has made

6. Does your centre plan to change the infection control practices in the coming 12 months?

- ◯ Yes
- ◯ No

7. Have you changed infection control practices for inpatient care?

- ◯ Yes
- ◯ No
- ◯ Not applicable

If yes, please specify the changes your centre has made

8. Has there been a change to the cleaning procedure?

- ◯ Yes
- ◯ No
- ◯ Not applicable

If yes, please specify the changes your centre has made

9. Has your centre implemented a mask wear policy?

- ◯ Yes
- ◯ No
- ◯ Not applicable

If yes, please specify the changes your centre has made

Physiotherapy infection control

General CF infection control guidelines

Please answer the following questions about infection control policies that existed in your centre as at 31st July 2017.

10. Are there infection control guidelines for managing people with CF that are followed by the physiotherapy team?

- ○ Yes
- ○ No

11. If yes, which guidelines do you follow (please tick all that apply)?

- ☐ Australian CF Infection Control Guidelines
- ☐ US CFF Infection Control Guidelines
- ☐ Local hospital infection control guidelines
- ☐ Other

If other, please specify

12. Are there specific infection control guidelines for managing people with CF and respiratory infection with (please tick all that apply):

- ☐ No Pseudomonas
- ☐ *Pseudomonas aeruginosa*
- ☐ MRSA
- ☐ *Burkholderia cepacia* complex
- ☐ *Mycobacterium abscessus* complex
- ☐ NTM species other than *M. abscessus* complex
- ☐ Other

If other, please specify

Physiotherapy infection control

Mask wearing

13. Has your hospital implemented that people with CF wear masks during hospital visits?

◯ Yes

◯ No

14. If yes, what type of mask are people with CF required to wear (please tick all that apply)?

☐ Surgical mask with ties

☐ Surgical mask with elastic ear loops

☐ N95 respirator

☐ Other

If other, please specify

15. If there is a mask policy in place, do you give instructions on (please tick all that apply):

☐ Where masks are located

☐ How to apply a mask

☐ How long to wear a mask

☐ When to change a mask

☐ How to dispose of the mask

☐ Hand hygiene after touching the mask

☐ Hand hygiene after removing the mask

16. Who applies the mask?

◯ Self

◯ Carer

◯ Healthcare worker

17. Are patients or carers trained in applying a mask?

◯ Yes

◯ No

18. If yes, how are they trained (please tick all that apply)?

- [ ] Signage
- [ ] One-on-one training
- [ ] Video
- [ ] Other

If other, please specify

19. During physiotherapy interventions, when is the mask required to be worn (please tick all that apply)?

- [ ] Exercise within own room
- [ ] Exercise in gym area
- [ ] During exercise testing (e.g. six minute walk test; shuttle test)
- [ ] When in transit outside patients' room

20. Are specific physiotherapy sessions conducted in the outpatient clinic for people with CF?

	Airway clearance	Induced sputum collection	Trial of inhaled antibiotics
All	○	○	○
No Pseudomonas	○	○	○
Pseudomonas aeruginosa	○	○	○
MRSA	○	○	○
Burkholderia cepacia complex	○	○	○
Mycobacterium abscessus complex	○	○	○
NTM species other than *M. abscessus* complex	○	○	○

If yes, please provide how these physiotherapy interventions are managed from an infection control perspective

21. Which personal protective equipment do physiotherapy staff wear during infectious outpatient clinics (please tick all that apply)?

	Gloves	Apron	Gown	Surgical mask with ties	Surgical mask with ear loops	N95 respirator	Other	None
No *Pseudomonas*	☐	☐	☐	☐	☐	☐	☐	☐
Pseudomonas aeruginosa	☐	☐	☐	☐	☐	☐	☐	☐
MRSA	☐	☐	☐	☐	☐	☐	☐	☐
Burkholderia cepacia complex	☐	☐	☐	☐	☐	☐	☐	☐
Mycobacterium abscessus complex	☐	☐	☐	☐	☐	☐	☐	☐
NTM species other than *M. abscessus* complex	☐	☐	☐	☐	☐	☐	☐	☐

If other, please specify

Physiotherapy infection control

Inpatient care

22. Where are airway clearance techniques performed for people with CF during hospital admissions (please tick all that apply)?

- [ ] Single room with ensuite
- [ ] Single room with shared bathroom
- [ ] Shared room
- [ ] Other

If other, please specify

23. Where is exercise performed for people with CF during hospital admissions (please tick all that apply)?

- [ ] Own hospital room
- [ ] Gym designated for people with CF only
- [ ] Communal gym (patients without CF may access the gym)
- [ ] Outdoors
- [ ] Other

If other, please specify

24. If exercise is performed in a gym, how are patients scheduled?

- ○ Individual sessions
- ○ With other people with CF and the same respiratory infection
- ○ With other people who do not have CF

25. In communal areas used by physiotherapists (such as gym, clinical rooms), do you have a washout period between patient consultations (please tick all that apply)?

	No washout	<30 minutes	30-60 minutes	>60 minutes
All CF patients	○	○	○	○
No *Pseudomonas*	○	○	○	○
Pseudomonas aeruginosa	○	○	○	○
MRSA	○	○	○	○
Burkholderia cepacia complex	○	○	○	○
Mycobacterium abscessus complex	○	○	○	○
NTM species other than *M. abscessus* complex	○	○	○	○

26. Do you have a separate gym for people with CF and the following infections (please tick all that apply):

- [ ] No *Pseudomonas*
- [ ] *Pseudomonas aeruginosa*
- [ ] MRSA
- [ ] *Burkholderia cepacia* complex
- [ ] *Mycobacterium abscessus* complex
- [ ] NTM species other than *M. abscessus* complex
- [ ] Other

If other, please specify

27. If gyms are not separate, are patients scheduled for gym attendance?

- ○ Yes
- ○ No

If yes, please specify

28. When is the gym equipment cleaned?

- ○ Between patients
- ○ Daily
- ○ Second daily
- ○ Weekly
- ○ Other

If other, please specify

29. Who cleans the gym equipment (please tick all that apply)?

- [ ] Physiotherapists
- [ ] Nursing staff
- [ ] Cleaning staff
- [ ] Other

If other, please specify

30. How is the gym equipment cleaned (please tick all that apply)?

- [ ] Alcohol-based wipes
- [ ] Biocidal wipes
- [ ] Sporicidal wipes
- [ ] Other

Please specify brands of cleaning products

31. Do you have special considerations of scheduling to see inpatients with certain infections (please tick all that apply)?

	Start of the day	End of the day	Separate day	Separate physiotherapist	Other
No *Pseudomonas*	☐	☐	☐	☐	☐
Pseudomonas aeruginosa	☐	☐	☐	☐	☐
MRSA	☐	☐	☐	☐	☐
Burkholderia cepacia complex	☐	☐	☐	☐	☐
Mycobacterium abscessus complex	☐	☐	☐	☐	☐
NTM species other than *M. abscessus* complex	☐	☐	☐	☐	☐

If other, please specify

32. Do you provide individual patient therapy equipment for people with CF during hospital admission (e.g. weights, exercise bikes, treadmills)?

- ○ Yes
- ○ No

33. When is the individual patient therapy equipment cleaned?

- ◯ Daily
- ◯ Second daily
- ◯ Weekly
- ◯ End of admission
- ◯ Other

If other, please specify

34. Who cleans the individual patient therapy equipment (please tick all that apply)?

- ☐ Physiotherapists
- ☐ Nursing staff
- ☐ Cleaning staff
- ☐ Other

If other, please specify

35. How is the individual patient therapy equipment cleaned (please tick all that apply)?

- ☐ Alcohol-based wipes
- ☐ Biocidal wipes
- ☐ Sporicidal wipes
- ☐ Other

Please specify brands of cleaning products

36. Are physiotherapy staff required to wear any personal protective equipment when treating inpatients (such as airway clearance techniques and exercise) (please tick all that apply)?

	Gloves	Apron	Gown	Surgical mask with ties	Surgical mask with elastic ear loops	N95 respirator	Other	None
No *Pseudomonas*	☐	☐	☐	☐	☐	☐	☐	☐
Pseudomonas aeruginosa	☐	☐	☐	☐	☐	☐	☐	☐
MRSA	☐	☐	☐	☐	☐	☐	☐	☐
Burkholderia cepacia complex	☐	☐	☐	☐	☐	☐	☐	☐
Mycobacterium abscessus complex	☐	☐	☐	☐	☐	☐	☐	☐
NTM species other than *M. abscessus* complex	☐	☐	☐	☐	☐	☐	☐	☐

If other, please specify

37. Do you have a stock of non-invasive ventilators (NIV) to use for patients with special infections (e.g. NTM, Bcc, MRSA) during physiotherapy treatment?

- ◯ Yes
- ◯ No

38. When is the NIV cleaned?

	Daily	Second daily	Weekly	End of patient use	Other
NIV unit	☐	☐	☐	☐	☐
Humidifier	☐	☐	☐	☐	☐
Tubing	☐	☐	☐	☐	☐
Mask	☐	☐	☐	☐	☐
Other	☐	☐	☐	☐	☐

For other NIV parts or other cleaning times, please specify.

39. Who cleans the NIV (please tick all that apply)?

- [ ] Physiotherapists
- [ ] Nursing staff
- [ ] Cleaning staff
- [ ] Other

If other, please specify

40. How is the NIV cleaned?

	Alcohol-based wipes	Biocidal wipes	Sporocidal wipes	Other
NIV unit	☐	☐	☐	☐
Humidifier	☐	☐	☐	☐
Tubing	☐	☐	☐	☐
Mask	☐	☐	☐	☐
Other	☐	☐	☐	☐

For other NIV parts please specify. Please provide brand names of cleaning products if known.

41. Are there any other infection control procedures, not highlighted in the above questions that you are required to undertake when treating people with certain infections?
